# 2,7-Bis­(trichloro­meth­yl)-1,8-naphthyridine^1^

**DOI:** 10.1107/S1600536810005234

**Published:** 2010-02-13

**Authors:** Hoong-Kun Fun, Suchada Chantrapromma, Annada C. Maity, Shyamaprosad Goswami

**Affiliations:** a X-ray Crystallography Unit, School of Physics, Universiti Sains Malaysia, 11800 USM, Penang, Malaysia; b Crystal Materials Research Unit, Department of Chemistry, Faculty of Science, Prince of Songkla University, Hat-Yai, Songkhla 90112, Thailand; c Department of Chemistry, Bengal Engineering and Science University’, Shibpur, Howrah, India 711 103

## Abstract

The complete mol­ecule of the title compound, C_10_H_4_Cl_6_N_2_, is generated by crystallographic twofold symmetry, with two C atoms lying on the rotation axis; the 1,8-naphthyridine ring is almost planar with an r.m.s. deviation of 0.0002 Å. In the crystal structure, the mol­ecules are stacked in an anti­parallel manner along [001]. Short Cl⋯Cl [3.3502 (4)] and Cl⋯N [3.2004 (11)–3.2220 (10) Å] contacts are observed in the crystal structure.

## Related literature

For bond-length data, see: Allen *et al.* (1987 ▶). For graph-set notation of hydrogen-bond motifs, see: Bernstein *et al.* (1995 ▶). For related structures, see: Fun *et al.* (2009 ▶); Wang *et al.* (2008 ▶). For background to the properties and applications of 1,8-naphthyridines, see: Braccio *et al.* (2008 ▶); Chen *et al.* (2001 ▶); Ferrarini *et al.* (1998 ▶; 2000 ▶). For the stability of the temperature controller used in the data collection, see: Cosier & Glazer (1986 ▶).
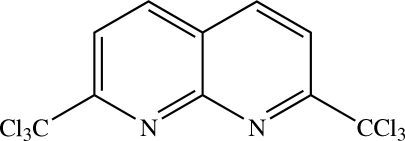


## Experimental

### 

#### Crystal data

C_10_H_4_Cl_6_N_2_
*M* *_r_* = 364.85
Monoclinic, 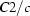

*a* = 19.9154 (4) Å
*b* = 6.5977 (1) Å
*c* = 10.5975 (2) Å
β = 111.483 (2)°
*V* = 1295.73 (4) Å^3^
*Z* = 4
Mo *K*α radiation
μ = 1.30 mm^−1^
*T* = 100 K
0.40 × 0.26 × 0.05 mm

#### Data collection

Bruker APEXII CCD diffractometer
Absorption correction: multi-scan (*SADABS*; Bruker, 2005 ▶) *T*_min_ = 0.624, *T*_max_ = 0.944
28872 measured reflections
4010 independent reflections
3136 reflections with *I* > 2σ(*I*)
*R*_int_ = 0.053

#### Refinement

*R*[*F* ^2^ > 2σ(*F* ^2^)] = 0.035
*wR*(*F* ^2^) = 0.089
*S* = 1.05
4010 reflections
91 parameters
All H-atom parameters refined
Δρ_max_ = 0.64 e Å^−3^
Δρ_min_ = −0.56 e Å^−3^

### 

Data collection: *APEX2* (Bruker, 2005 ▶); cell refinement: *SAINT* (Bruker, 2005 ▶); data reduction: *SAINT*; program(s) used to solve structure: *SHELXTL* (Sheldrick, 2008 ▶); program(s) used to refine structure: *SHELXTL*; molecular graphics: *SHELXTL*; software used to prepare material for publication: *SHELXTL* and *PLATON* (Spek, 2009 ▶).

## Supplementary Material

Crystal structure: contains datablocks global, I. DOI: 10.1107/S1600536810005234/hb5307sup1.cif

Structure factors: contains datablocks I. DOI: 10.1107/S1600536810005234/hb5307Isup2.hkl

Additional supplementary materials:  crystallographic information; 3D view; checkCIF report

## Figures and Tables

**Fig. 1.**
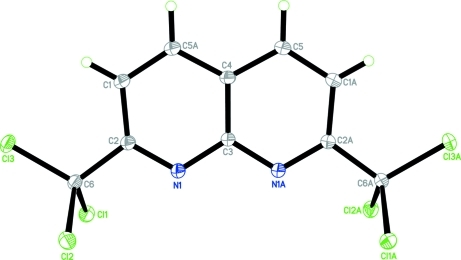
The molecular structure of (I) showing 50% probability displacement ellipsoids. Atoms with suffix A were generated by symmetry code -x, y, -1/2 - z.

**Fig. 2.**
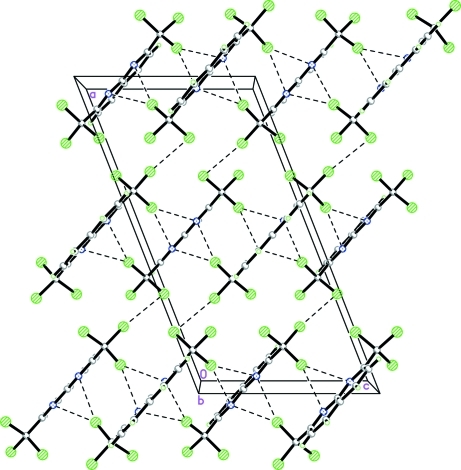
The crystal packing of the title compound viewed along the b axis, N···Cl and C···.Cl short contacts are shown as dashed lines.

## supplementary crystallographic information

### Comment

The substituted 1,8-naphthyridine compounds have been studied for their chemical and biological activities for a long time. They show various biological activities such as antibacterial (Chen *et al.*, 2001; Ferrarini *et al.*, 1998), anti-inflammatory (Braccio *et al.*, 2008) as well as antihypertensive (Ferrarini *et al.*, 2000) properties. Trichloromethyl-substituted heterocyclic compounds are of great importance due to their broad spectrum biological activities. These interesting properties prompt us to synthesise the title compound (I) and its crystal structure was reported.

The asymmetric unit of the title molecule (Fig. 1), C_10_H_4_Cl_6_N_2_, contains one half-molecule with two shared C atoms (C3 and C4) lying on a twofold rotation axis. The 1,8-naphthyridine ring is planar with the r.m.s. deviation of 0.0002 (2) Å. One Cl atom (Cl3) of the trichloromethyl substitutent is co-planar with the 1,8-naphthyridine ring which can be indicated by the torsion angle C1–C2–C6–Cl3 = 1.85 (14) Å whereas the other two Cl atoms are in the (+)-anti-clinal and (-)-anti-clinal configurations with the torsion angles C1–C2–C6–Cl1 = 122.04 (10)° and C1–C2–C6–Cl2 = -119.02 (10)°, respectively. The C1—H1···Cl3 intramolecular interaction (Table 1) generates S(5) ring motif (Bernstein *et al.*, 1995). The bond distances are of normal values (Allen *et al.*, 1987) and are comparable with related structures (Fun *et al.*, 2009; Wang *et al.*, 2008).

In the crystal structure (Fig. 2), the non-covalent interactions play a significant role in the three-dimensional supramolecular architecture in which the molecules are stacked in an antiparallel manner along the [0 0 1] direction and the neighbouring molecules are interlinked by C—Cl···Cl interactions into polymeric chains along the [0 1 0] direction. The molecules are also consolidated by Cl···Cl [3.3502 (4)Å] and Cl···N [3.2004 (11)–3.2220 (10) Å] short contacts. π···π interactions were observed with the distances of *Cg*_1_···*Cg*_1_ = 4.2360 (6) Å (symmetry code: -*x*, 1 - *y*, 2 - *z*) and *Cg*_2_···*Cg*_2_ = 4.2360 (6) Å (symmetry code: -*x*, 1 - *y*, 1 - *z*): *Cg*_1_ and *Cg*_2_ are the centroids of C1–C4/C5A/N1 and C1A–C2A/C3–C5/N1A, respectively. All these interactions connect the molecules into a three-dimensional supramolecular network.

### Experimental

A mixture of *N*-chlorosuccinimide (500 mg, 4.5 mmol) and triphenylphosphine (500 mg, 4.2 mmol) was moistened with CCl_4_ (60 ml) in a round bottom flask and stirred at room temperature for 25 min. A solution of 2,7-dimethyl-1,8-naphthyridine (0.9 g, 5.25 mmol) was added to the suspension and the reaction mixture was stirred and heated under reflux for 7 hr. The solution was cooled and filtered. The evaporated filtrate was washed with saturated aqueous Na_2_CO_3_ and extracted repeatedly with CHCl_3_. Drying over anhydrous Na_2_SO_4_, the solvent was removed under reduced pressure. The crude product was purified with SiO_2_ chromatography (eluted with 1% ethylacetate in petroleum ether) to give the title compound as a white crystalline solid. Colorless slabs of (I) were recrystalized from CH_2_Cl_2_:hexane (1:10, *v*/*v*) by the slow evaporation of the solvent at room temperature after a week.

### Refinement

H atoms were located in a difference maps and refined isotropically. The highest residual electron density peak is located at 1.72 Å from H6 and the deepest hole is located at 0.65 Å from Cl2.

### Crystal data

**Table:**

C_10_H_4_Cl_6_N_2_	*F*(000) = 720
*M**_r_* = 364.85	*D*_x_ = 1.870 Mg m^−^^3^
Monoclinic, *C*2/*c*	Mo *K*α radiation, λ = 0.71073 Å
Hall symbol: -C 2yc	Cell parameters from 4010 reflections
*a* = 19.9154 (4) Å	θ = 2.2–40.0°
*b* = 6.5977 (1) Å	µ = 1.30 mm^−^^1^
*c* = 10.5975 (2) Å	*T* = 100 K
β = 111.483 (2)°	Slab, colorless
*V* = 1295.73 (4) Å^3^	0.40 × 0.26 × 0.05 mm
*Z* = 4	

### Data collection

**Table:**

Bruker APEXII CCD diffractometer	4010 independent reflections
Radiation source: sealed tube	3136 reflections with *I* > 2σ(*I*)
graphite	*R*_int_ = 0.053
φ and ω scans	θ_max_ = 40.0°, θ_min_ = 2.2°
Absorption correction: multi-scan (*SADABS*; Bruker, 2005)	*h* = −36→34
*T*_min_ = 0.624, *T*_max_ = 0.944	*k* = −11→11
28872 measured reflections	*l* = −18→19

### Refinement

**Table:**

Refinement on *F*^2^	Primary atom site location: structure-invariant direct methods
Least-squares matrix: full	Secondary atom site location: difference Fourier map
*R*[*F*^2^ > 2σ(*F*^2^)] = 0.035	Hydrogen site location: inferred from neighbouring sites
*wR*(*F*^2^) = 0.089	All H-atom parameters refined
*S* = 1.05	*w* = 1/[σ^2^(*F*_o_^2^) + (0.0403*P*)^2^ + 0.889*P*] where *P* = (*F*_o_^2^ + 2*F*_c_^2^)/3
4010 reflections	(Δ/σ)_max_ = 0.002
91 parameters	Δρ_max_ = 0.64 e Å^−^^3^
0 restraints	Δρ_min_ = −0.56 e Å^−^^3^

### Special details

**Table:**

Experimental. The crystal was placed in the cold stream of an Oxford Cryosystems Cobra open-flow nitrogen cryostat (Cosier & Glazer, 1986) operating at 120.0 (1) K.
Geometry. All e.s.d.'s (except the e.s.d. in the dihedral angle between two l.s. planes) are estimated using the full covariance matrix. The cell e.s.d.'s are taken into account individually in the estimation of e.s.d.'s in distances, angles and torsion angles; correlations between e.s.d.'s in cell parameters are only used when they are defined by crystal symmetry. An approximate (isotropic) treatment of cell e.s.d.'s is used for estimating e.s.d.'s involving l.s. planes.
Refinement. Refinement of *F*^2^ against ALL reflections. The weighted *R*-factor *wR* and goodness of fit *S* are based on *F*^2^, conventional *R*-factors *R* are based on *F*, with *F* set to zero for negative *F*^2^. The threshold expression of *F*^2^ > σ(*F*^2^) is used only for calculating *R*-factors(gt) *etc*. and is not relevant to the choice of reflections for refinement. *R*-factors based on *F*^2^ are statistically about twice as large as those based on *F*, and *R*- factors based on ALL data will be even larger.

### Fractional atomic coordinates and isotropic or equivalent isotropic displacement parameters (Å^2^)

**Table:**

	*x*	*y*	*z*	*U*_iso_*/*U*_eq_	
Cl1	0.090117 (15)	0.01827 (4)	1.12884 (3)	0.01585 (6)	
Cl2	0.194324 (15)	0.02817 (4)	0.99732 (3)	0.01645 (6)	
Cl3	0.202697 (15)	0.31574 (4)	1.20815 (3)	0.01848 (6)	
N1	0.04657 (5)	0.21807 (14)	0.85379 (9)	0.01358 (15)	
C1	0.09721 (6)	0.53166 (17)	0.96675 (12)	0.01599 (18)	
C2	0.09266 (6)	0.31794 (16)	0.95656 (11)	0.01302 (16)	
C3	0.0000	0.3281 (2)	0.7500	0.0124 (2)	
C4	0.0000	0.5430 (2)	0.7500	0.0136 (2)	
C5	−0.05049 (6)	0.64457 (17)	0.63741 (11)	0.01611 (18)	
C6	0.14272 (6)	0.17959 (16)	1.06796 (10)	0.01333 (16)	
H6	−0.0513 (10)	0.788 (3)	0.6355 (18)	0.021 (4)*	
H1	0.1292 (10)	0.598 (3)	1.038 (2)	0.024 (5)*	

### Atomic displacement parameters (Å^2^)

**Table:**

	*U*^11^	*U*^22^	*U*^33^	*U*^12^	*U*^13^	*U*^23^
Cl1	0.01798 (11)	0.01518 (11)	0.01362 (10)	−0.00263 (8)	0.00487 (8)	0.00164 (8)
Cl2	0.01578 (11)	0.01700 (12)	0.01533 (11)	0.00202 (8)	0.00425 (8)	0.00040 (8)
Cl3	0.02005 (12)	0.01629 (11)	0.01330 (11)	−0.00383 (9)	−0.00076 (8)	−0.00181 (8)
N1	0.0155 (4)	0.0114 (3)	0.0115 (3)	−0.0004 (3)	0.0021 (3)	0.0002 (3)
C1	0.0186 (4)	0.0110 (4)	0.0152 (4)	−0.0022 (3)	0.0025 (4)	−0.0012 (3)
C2	0.0145 (4)	0.0113 (4)	0.0123 (4)	−0.0008 (3)	0.0038 (3)	0.0001 (3)
C3	0.0137 (5)	0.0101 (5)	0.0117 (5)	0.000	0.0027 (4)	0.000
C4	0.0173 (6)	0.0095 (5)	0.0132 (5)	0.000	0.0045 (5)	0.000
C5	0.0198 (5)	0.0104 (4)	0.0155 (4)	0.0011 (3)	0.0033 (4)	0.0009 (3)
C6	0.0148 (4)	0.0122 (4)	0.0113 (4)	−0.0015 (3)	0.0028 (3)	−0.0007 (3)

### Geometric parameters (Å, °)

**Table:**

Cl1—C6	1.7725 (11)	C2—C6	1.5358 (15)
Cl2—C6	1.7827 (11)	C3—N1^i^	1.3602 (12)
Cl3—C6	1.7723 (10)	C3—C4	1.418 (2)
N1—C2	1.3156 (14)	C4—C5	1.4160 (13)
N1—C3	1.3602 (12)	C4—C5^i^	1.4160 (13)
C1—C5^i^	1.3736 (16)	C5—C1^i^	1.3737 (16)
C1—C2	1.4145 (15)	C5—H6	0.95 (2)
C1—H1	0.90 (2)		
			
C2—N1—C3	117.68 (10)	C5—C4—C3	118.24 (7)
C5^i^—C1—C2	118.28 (10)	C5^i^—C4—C3	118.24 (7)
C5^i^—C1—H1	118.1 (14)	C1^i^—C5—C4	118.91 (11)
C2—C1—H1	123.6 (14)	C1^i^—C5—H6	121.6 (11)
N1—C2—C1	124.62 (10)	C4—C5—H6	119.5 (11)
N1—C2—C6	113.48 (9)	C2—C6—Cl3	113.05 (7)
C1—C2—C6	121.90 (9)	C2—C6—Cl1	109.44 (7)
N1—C3—N1^i^	115.46 (13)	Cl3—C6—Cl1	107.82 (6)
N1—C3—C4	122.27 (6)	C2—C6—Cl2	108.79 (7)
N1^i^—C3—C4	122.27 (6)	Cl3—C6—Cl2	108.72 (6)
C5—C4—C5^i^	123.51 (14)	Cl1—C6—Cl2	108.95 (6)
			
C3—N1—C2—C1	0.00 (16)	N1^i^—C3—C4—C5^i^	179.98 (8)
C3—N1—C2—C6	−179.95 (8)	C5^i^—C4—C5—C1^i^	180.00 (13)
C5^i^—C1—C2—N1	−0.02 (19)	C3—C4—C5—C1^i^	0.00 (13)
C5^i^—C1—C2—C6	179.92 (11)	N1—C2—C6—Cl3	−178.20 (8)
C2—N1—C3—N1^i^	−179.98 (11)	C1—C2—C6—Cl3	1.85 (14)
C2—N1—C3—C4	0.02 (11)	N1—C2—C6—Cl1	−58.01 (11)
N1—C3—C4—C5	179.98 (8)	C1—C2—C6—Cl1	122.04 (10)
N1^i^—C3—C4—C5	−0.02 (8)	N1—C2—C6—Cl2	60.93 (11)
N1—C3—C4—C5^i^	−0.02 (8)	C1—C2—C6—Cl2	−119.02 (10)

Symmetry codes: (i) −*x*, *y*, −*z*+3/2.

### Hydrogen-bond geometry (Å, °)

**Table:**

*D*—H···*A*	*D*—H	H···*A*	*D*···*A*	*D*—H···*A*
C1—H1···Cl3	0.90 (2)	2.63 (2)	3.0085 (12)	106.0 (14)
